# A Novel Scheme for Controller Selection in Software-Defined Internet-of-Things (SD-IoT)

**DOI:** 10.3390/s22093591

**Published:** 2022-05-09

**Authors:** Jehad Ali, Byeong-hee Roh

**Affiliations:** 1 Department of Computer Engineering, Ajou University, Suwon 16499, Korea; jehadali@ajou.ac.kr; 2 Department of AI Convergence Network, Ajou University, Suwon 16499, Korea

**Keywords:** SDN, performance evaluation, controller, OpenFlow, ANDP

## Abstract

The software-defined networking (SDN) standard decouples the data and control planes. SDN is used in the Internet of Things (IoT) due to its programmability, central view and deployment of innovative protocols, and is known as SD-IoT. However, in SD-IoT, controller selection has never been studied. Controllers control the network and react to dynamic changes in SD-IoT. As sensors communicate frequently with the controller in SD-IoT, there is a degradation in performance with scalability and an increase in flow requests. Hence, the controller performance and selection are critical for SD-IoT. However, one controller’s support for certain functions is high while another’s is poor. There are various SD-IoT controllers, and choosing the best one might be a multi-criteria choice. An analytical network decision making process- (ANDP) based technique is employed here to identify feature-based optimal controllers in SD-IoT. The experimental analysis quantifies the high-weight controller from the feature-based comparison. An ANDP-based feature-based controller selection strategy is suggested, which selects the controller with the best feature set first, before comparing performance. This paper’s main contribution is to evaluate the ANDP for SD-IoT controller selection based on its features and performance validation in the SD-IoT environment. The simulation results suggest that the proposed controller outperforms the controller selected with previous schemes. Choosing an optimal controller in SD-IoT reduces the delay in both normal and heavy traffic scenarios. The suggested controller also increases throughput while using the central processing unit (CPU) efficiently and reduces the recovery latency in case of failures in the network.

## 1. Introduction

The Internet-of-Things (IoT) [1] and 5G have made software-defined networking (SDN) [2] an excellent alternative because of its revolutionary characteristics of centralized view, flexible administration, and network function virtualization (NFV) support. Similarly, megacorp IT corporations like Amazon, Facebook and Google have already implemented SDN and are helping to standardize SDN protocols and architecture via the open networking foundation (ONF) [3]. Google launched a well-known initiative dubbed B4 [4] to link its foreign data centers using novel SDN capabilities. This eliminated vendor dependence on its hardware, enabled centralized control, and increased network management flexibility via well-known application programming interfaces (APIs). Consequently, this has led to cost savings, better efficiency, and the rapid deployment of new services.

**Figure 1:**
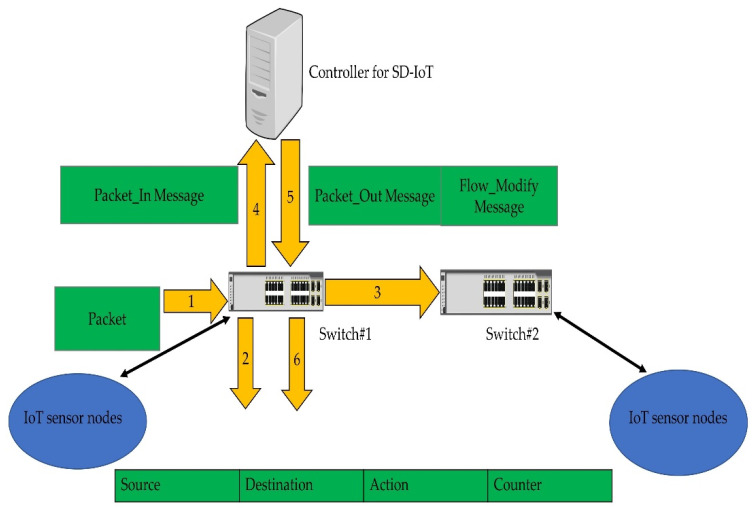
Sensor nodes generating traffic in SD-IoT [22].

The relevance of the SDN controller has increased as a consequence of the unique centralized management model presented by SDN. In contrast to decentralized and traditional networks, the SDN provides a global picture of the network beneath its control, via the centralized management through the controller, allowing operators to program data plane devices and apply rules from a single location. Apart from the many benefits of SDN, its modelling, assessment, and testing pose a number of problems. One of the difficulties is deciding on the best SDN controller, since each controller has many supporting characteristics. The controller’s mandatory function in a standard SDN mandates the selection of an optimal controller. Figure 1 depicts a software-defined Internet-of-Things (SD-IoT) scenario in which the SDN switch forwards the arrived packet according to the flow rules if the rules already exist in the flow table (step 1, 2, and 3), or sensor nodes submit flow requests to the central controller as Packet-In messages when there is no flow rule in the associated switch to the sensors. i.e., known as a table miss in SDN i.e., denoted with step 1, and 4. The controller then finds the destination and sends a Packet-Out message to update the flow rules (step 5 and 6). As a result, the controller’s performance degrades, and the latency and other performance characteristics such as throughput and CPU utilization suffer. Hence, in SD-IoT, an appropriate controller is required to accommodate a high number of sensor nodes.

Due to the controller’s crucial role in SDN, several studies have compared SDN controllers. These studies employ Cbench [5] or Mininet [6] to choose a controller from a group based on SDN performance. The study by [7] examined five SDN controllers: Ryu, Nox, Beacon, Pox and Floodlight [8,9,10,11,12], in both throughput and latency modes. Mul and Maestro were also included in a comparable performance comparison study [13,14]. The authors employed Cbench to measure each system’s throughput and delay metrics.

In [15], the authors built a network design in Mininet and then ran it on the controller to analyse SDN controller performance. Then they used Iperf and Ping to compare TCP performance and latency. They used a tree network simulating 16 hosts with a fanout of four, where the authors evaluated four controllers: Odl [16], Pox, Onos [17], and Ryu [18], and examined the same performance parameters with three hosts and one switch. In a study [19,20,21], the authors examined the performance of SDN controllers and various benchmarking methods. These studies used Cbench or Mininet to choose controllers based on performance indicators (latency and throughput). Throughout the studies in the selection process, researchers have paid little attention to the controllers’ supporting characteristics. Assistive features like OpenFlow [3] support and flow request management are examples of supporting features. As a consequence, this article examines these controllers’ features and their relevance for SD-IoT performance evaluation.

To the best of our knowledge, this is the first research that assesses the performance of SD-IoT controllers chosen considering their features in SD-IoT. We present a hybrid strategy towards SDN controller selection for SD-IoT in this research using the features-based ranking of the controllers relevant for SD-IoT, followed by the performance evaluation of the high weight controller. The ANDP is used in the first round to pick a controller. Moreover, the top controller is evaluated through quantitative performance based on the high weights. The quantitative study compares delay, throughput, CPU usage, and link failure robustness of the controllers with controllers derived from benchmark models using Mininet. The controller’s performance is validated and evaluated in the Mininet. We previously examined the performance of the Ryu and Pox controllers in several topologies, [23] i.e., linear, single, dumbbell, Tree, and data centre networks (DCNs) were among the topologies used for evaluation. In this paper, we propose a mathematical decision-making framework by first calculating the optimal controller in terms of its features that enhance the performance of SD-IoT using an ANDP model and then we validate it through a performance investigation of the controller in SD-IoT leveraging the SDN simulation tool Mininet. Moreover, we compare our proposed model with previous benchmark schemes [24,25] and evaluate it with several experiments.

The rest of the paper is organized as follows. Section 2 describes related works and the contributions of our paper. Section 3 discusses problem formulation. Our proposed model for SDN controller ranking in SD-IoT leveraging the ANDP approach and the feature-based grouping as a pre-processing step is illustrated in Section 4. In Section 5, the performance of the proposed ANDP framework and previous studies is evaluated for SD-IoT using Mininet. Finally, Section 6 includes concluding remarks based on our findings from the mathematical ANDP scheme.

## 2. Related Works

Various techniques for SDN controller selection have been proposed in the literature. These techniques may be divided into three types. The first category includes evaluating controllers by considering only their features. In contrast, the second type relies on the performance evaluation, and the third on a hybrid basis. The hybrid technique chooses the best controller by consolidating the feature and performance comparisons. These methods are investigated in the following literature studies.

The research experiments reported in [7,15,19,20] merely compare SDN controller performance. Performance-based methods exclusively examine performance while ignoring SDN controller characteristics. Second, these methodologies consider performance in SDN infrastructure using Mininet or by constructing virtual nodes and switches in Cbench. As a result, the SD-IoT situation is not considered in their experiments since the networks are simulated only for virtual resources. The studies [26,27,28,29,30] on controller features give a comparison of various controllers in terms of the supporting functions that they provide. The platforms REST API, clustering, and OpenFlow support are examples of supporting features. The purpose of all techniques is to find the best SDN controller. Nevertheless, approaches centered only on the features ignore the performance investigation for controllers in SDN.

The researchers in [31] presented a comparative evaluation of four SDN controllers using a hybrid method. The authors chose two controllers from the controller feature table using a heuristic choice. They identified nine features that these controllers provided and chose two controllers based on an examination of the features table. They then used Cbench to analyse the performance of these controllers in throughput and latency mode. Hence, they only examined the feature table, and their analysis did not offer a clear rating of these controllers. As a result, in using their technique, exact selection is impossible. Second, they have not taken into account the performance comparison in SD-IoT. In [32], the researchers examined SDN controllers, i.e., Maestro, Beacon, Odl, Ryu, Nox, and Libfluied Raw [33] by increasing the threads number and switches in both throughput and delay modes. A comparable performance evaluation looked at four controllers: Ryu, Floodlight, Onos, and Odl. This research is demonstrated in [34], and the authors ran Cbench to calculate the throughput and latency for each controller.

In [35], a qualitative and quantitative comparison of five SDN controllers, Trema [36], Ryu, Odl, Floodlight, and Onos, was undertaken evaluating aerial networks. First, a qualitative analysis of these controllers was conducted in terms of two features, i.e., clustering capacity and handling state information. The state controlling information for five controllers was organized as a table to investigate how each controller obtains and stores the information about the state of the aerial network, as well as the condition of this information in the event of failure for a switch or SDN controller. Specifically, the authors were interested in whether the controller will restock this information after a previously stored state or re-generate the status of the network. Correspondingly, the data about each controller’s clustering technique was tabulated to see whether these controllers assist clustering and how various controllers communicate information about the cluster they are handling. The two top significant controllers in their research were chosen based on the two features that met the criterion of an aerial network. A performance assessment was carried out using a Mininet-simulated experimental scenario. Their controller selection approach, on the other hand, was built on a heuristic preference, which results in cognitive overload with scalability in the SDN controllers and their features.

Multi-criteria decision-making (MCDM) is a decision-making methodology in which a set of criteria is used to pick one of multiple possibilities [37] or alternatives. It has been extensively employed in a variety of domains, including software development for strategy selection [38], natural resource management [39], for network selection comprises of heterogeneous networks [40], etc. Different techniques, such as the analytical hierarchy process (AHP) [41] and others, are used for the selection process based on several criteria to achieve a desired goal. The authors in [42] advocated utilizing an MCDM approach such as AHP to pick SDN controllers. The research took into account 10 controllers and features in order to pick the controller established according to its characteristics. However, they did not undertake a quantitative experimental comparison of these controllers. Moreover, their study provides no specifics on the technique they utilized. The feedback from the other cluster parts, as well as the dependencies between them, are taken into account by the ANDP. The AHP lacks a method for feedback and component dependence [41] in making decisions, while the ANDP covers the feedback and dependence among elements upon which the decision will take place.

The researchers in [25,43] illustrated a hybrid strategy for controller selection by evaluating the features as well as the performance of the controllers by using a combination of AHP and a technique for order of preference by similarity to ideal solution (TOPSIS) [43] and an entropy-based TOPSIS (EB-TOPSIS) [25] framework. The authors selected the Floodlight controller via feature evaluation. Furthermore, the features related to IoT in SDN have not been considered in the selection process. Moreover, the scenarios are demonstrated with a smaller number of nodes that cannot reflect an IoT scenario.

Similarly, in [25], the authors describe a hybrid method of controller selection based on AHP. The prioritized three controllers computed from AHP were examined for performance testing using Cbench in that research; however, they did not analyse the performance for SD-IoT. The mathematical specifics of the authors’ technique were not provided. The input from the alternatives was not taken into account in AHP. As a result, AHP ignores this feedback feature and concentrates only on the selection criteria. Another disadvantage of AHP is that the criteria (also known as features of controllers) was considered independently utilizing AHP, making it impossible to make a precise decision. The ANDP was utilized in [44] to modify risk variables in megaprojects utilizing the risk index. Similarly, Shah Nazir et al. [45] applied it to pick software components using quality as a criterion. Furthermore, the ANDP has been utilized for the networking of wireless sensors to select an ideal cluster head [46]. Hence, we conclude that the ANDP technique can be used to analyse systems together with complex behaviour and structure. As the complexity of various systems has enhanced their interdependence, the research of interdependent methods is a critical challenge in network systems [47]. Moreover, ANDP [48,49,50] is a mathematically supported model-based instrument in the decision-making process that is based on a number of factors. Hence, we make a mathematical model for controller selection in SD-IoT in this paper leveraging the features necessary in an IoT environment.

### Research Gap, and Contributions of the Proposed Scheme

In related works, the authors have not investigated the features for SD-IoT, and neither does there exist a comprehensive study regarding a hybrid mechanism considering feature significance and realistic SD-IoT experimental evaluations. However, our suggested controller selection technique is based on a qualitative and quantitative examination of SDN controllers for SD-IoT. First, we determined the characteristics of the controllers for the IoT environment. We then used ANDP to determine the high weight SD-IoT controller. By computing weights for each controller, the ANDP ranks the controllers with the best feature set for SD-IoT among others. Furthermore, the quantitative assessment of a high-weight ranked controller is carried out in Mininet through multiple simulations. The technique for selecting the best SDN controller is outlined below:

1. A list of SDN controllers is identified, along with the functionalities required for an IoT environment in SDN.
2. We then perform feature pre-processing to determine the support level of each feature in the specific controller.
3. We formulate the problem of the controller selection in SD-IoT with ANDP to select the controller with high-weight value for SD-IoT.
4. Finally, we evaluate the performance of the controller computed through ANDP via several simulations in a standard SDN simulation tool prevalent for state-of-the-art SDN research.
5. Finally, the performance of the proposed ANDP controller for SD-IoT is compared through a controller computed with AHP [24], and EB-TOPSIS [25] schemes in the previous research for controller selection.

The study adds to the controller selection issue by exploiting the properties of the SDN controller using ANDP for SD-IoT when comparing SDN controllers. Second, the performance of the chosen controller with ANDP and AHP is compared in SD-IoT topologies. Finally, the performance measurement is carried out in Mininet, an SDN environment emulator. Moreover, a comprehensive performance comparison evaluation is conducted with previous methods [24,25].

## 3. Problem Formulation

In SDN, the performance is directly dependent on the controller. As a result, selecting the best SDN controller will guarantee optimal network usage, hence enhancing the quality of service (QoS) in SD-IoT. Each controller offers a number of characteristics, including OpenFlow, platform compatibility, and south and northbound interfaces, as illustrated in Table 1. The SDN controllers are shown in Table 2. These are significant SDN controllers, as recent studies have considered it for comparative analysis [42,43] because of the support for the new features (shown in Table 1) which are important in SDN. Similarly, each controller supports a distinct set of platforms. For example, Pox sustains Mac, Linux, and Windows, but Trema exclusively supports Linux. Furthermore, each controller provides varying levels of scalability, flow request management, and energy support. Similarly, each controller supports a distinct OpenFlow version (e.g., 1.0, or 1.1, or 1.2 etc.).

**Figure 2:**
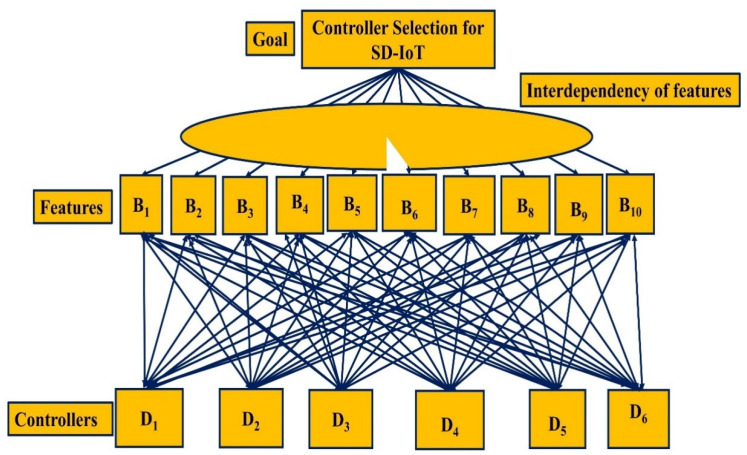
The ANDP model for controller selection in SD-IoT.

The controller is so important in SDN that it should be chosen with care. An MCDM issue is the selection of a controller based on multiple attributes. The ANDP is commonly utilized in multi-criteria decision-making issues where alternate feedback and interdependence among criteria or features are taken into account. The ANDP algorithm will choose the best controller from a group of controllers before deploying it. Figure 2 depicts the ANDP model for paired comparisons in SD-IoT controller selection. It shows the ranking model of the ANDP, which consists of a features cluster i.e., the top one, and the alternatives cluster i.e., the bottom one. Moreover, another line in the form of the circle shows the interdependency among features. In addition, the arrows between the features and alternative cluster denote the pairwise comparisons. Section 4 describes the detailed approach for selecting the best controller for SD-IoT using the ANDP model with mathematical expressions.

## 4. Proposed Mathematical Model Using ANDP for Controller Selection in SD-IoT

As illustrated in Figure 2, the ANDP MCDM issue is constructed by first describing the aim or objective, then specifying the parameters for criterion, and then identifying the alternatives or controllers under evaluation. Herein, our goal in this research is to find the best SDN controller for SD-IoT based on the 10 characteristics listed in Table 1. Equations (1) and (2) reflect the criterion for SD-IoT and controllers. B represents the accessible features provided by the various SDN controllers, and D represents the choices from Equation (2). Herein, the ANDP strategy considers the additional features i.e., B_7_, B_8_, and B_10_ of the IoT environment in addition to the features significant for the general SDN. The IoT in the next generation networks (5G and beyond) deals with the large number of sensor nodes [51]. Hence, the flow requests generated by the controller shall be large. Furthermore, the support of the B_7_ feature in the SDN controllers is important in handling a huge number of flow requests generated by the data plane sensor nodes. In addition, with the number of sensor nodes increasing the scalability feature, B_8_ is also significant in the controllers for the SD-IoT. Furthermore, with a large number of flow requests and scalability, the B_10_ feature of energy management plays an important role in the controllers. Hence, the ANDP approach employs these features. Moreover, we make an evaluation of the top ranked controller embedded with these features in the IoT environment through Mininet emulation, i.e., the data plane for which the ANDP controller is to be tested is from the IoT sensor nodes. In contrast to the ANDP strategy, the analytical network process (ANP) [52] considers features for the general SDN. The detailed description of the ANDP controller selection for SD-IoT is given in the following subsections.

(1) $$B=\left\{B_{1},B_{2},B_{3},\;\ldots ,{\;B}_{N}\right\}$$

(2) $$D=\left\{D_{1},D_{2},D_{3},\;\ldots ,{\;D}_{n}\right\}$$

### 4.1. Features Pre-Processing for SD-IoT

The research in [52,53,54] provides the ten critical aspects that should be examined when selecting a controller as a criterion. As a result, we agree that all of these characteristics are necessary for the controller selection technique. However, since controllers are constantly changing, we took into account the most recent information in relation to these aspects from documentation concerning a controller and studies in [24,25,43], Moreover, we considered the scalability, energy management, and flow request handling features necessary for IoT.

These critical qualities are employed and taken into account in the optimal controller selection process in SD-IoT utilizing ANDP. As a result, classifying these characteristics identifies the value of a feature in each controller.

A controller’s features are classified into two types: (1) ordinal and (2) categorical. The ordinal features come up with an inherent listing, but the categorical controller features do not have an inherent ordering. The categorization of the feature set provides a clear understanding of the extent of support for that feature in the controller. We classified the characteristics as G_1_-G_4_, with G_1_ indicating extremely low support and G_4_ denoting very strong support. G_2_ indicates medium support, but G_3_ only reveals strong support. For example, D_4_ and D_6_ only support OpenFlow v1.0, hence they are retained in G_1_ for this feature (B_1_), as indicated in Table 3. D_1_ support is medium, and D_2_ and D_3_ support v1.0,1.1,1.3, so they are preserved in G_3_, and D_5_ supports higher versions of OpenFlow, i.e., 1.5, thus it is kept in G_4_.

Similarly, controllers are classified according to B_9_, or the platform on which they are supported. D_2_, D_3_, and D_4_ have support on three platforms, namely Linux, Mac, and Windows, and hence are classified as G_3_. The D_1_, D_5_, and D_6_ are only supported by one platform, Linux, so they are awarded a G_1_ classification. B_7_, B_8_, and B_10_ demonstrate flow handling, scalability, and energy management capabilities. D_3_ has a high degree of support for these features, hence it is assigned the G_4_ level. Similarly, additional ordinal characteristics are classified in each controller based on their amount of support. A controller may or may not offer REST API, open stack networking, or clustering as an example of a normal categorical functionality. As a result, these qualities (B_3_, B_4_, and B_5_) do not have an inherent ordering and are represented in Table 3 with a yes or no. Before creating the comparison matrix, feature classification is performed as a pre-processing step.

### 4.2. The Comparison of Controllers Regarding Their Features for SD-IoT

Alternatives (controllers) are pairwise compared regarding every feature. The general structure of the matrix for pairwise comparisons is shown in matrix (3). First, the alternatives are compared using (3) by considering their B1 feature in every controller. The values are incorporated in (3). We used a five-level scale for SD-IoT controller selection with ANDP rather than the nine-levels scale used by ANP [53]. Herein, we illustrate the details of the five-levels employed by the ANDP.

1. A value of 1 is assigned in the comparison matrix if the two features have an equal importance in the controllers.
2. However, if one feature is moderately more important than the other, then a value of three indicates the level of importance.
3. Moreover, a feature that is significantly important with respect to other controllers is denoted with a number 5 in the comparison matrix.
4. Furthermore, a feature showing a significantly important level in a controller compared to the other controllers is given a value of 7.
5. Finally, the extremely important level of a feature in a controller compared to the others is represented with a value of 9.

The resultant inserted values for B1 are shown in matrix (4). The nominator and denominator values identify the relative significance of row and column elements (controllers), respectively. In matrix (4), D_1_ is compared with D_2_, D_3_, D_4_, D_5_ and D_6_ considering the B_1_ criterion. The matrix shows that D_1_ is of equal importance with itself i.e., $a_{11}$ = 1. D_2_ and D_3_ are therefore moderately more important than D_1_. i.e., $a_{\left(1,2\right)}=a_{\left(1,3\right)}=\frac{1}{3}$. D_1_ is moderately more important than D_4_ and D_6_ e.g., $a_{16}$ = 3 shows that the controller in this row (D_1_) is rather more important than the controller in the subsequent column (D_6_). $a_{\left(1,5\right)}=\frac{1}{5}$ reveals that D_5_ is significantly more important than D_1_. Correspondingly, the values are covered for D_2_, D_3_, D_4_, D_5_ and D_6._

Matrix (4) is the outcome of all judgments of the controllers for the B_1_ feature. According to matrix (4), each column’s total values are added together, and each individual value is divided by the sum of the column’s total values (5). The final product is a normalized matrix, as seen in matrix (5). The eigenvector $\hat{H}$_1_ is represented in (6). The next step is to obtain the U and K values to see whether the judgments made while creating the pairwise matrix are consistent. However, the consistency measure (CM) vector must be calculated before the consistency analysis can be performed.

Consistency Measure (CM): CM is denoted as a vector, which is a prerequisite for obtaining U and K. It is represented in Equation (8). The $\hat{H}$ and ${\hat{h}}_{i}$ identify the eigen vector as well as the corresponding element of an Eigen vector as denoted in Equation (8). Equation (7) denotes that the values of rows (R_j_) of the comparison matrix and $\hat{H}$ are multiplied and then divided by the matrix element ${\hat{h}}_{i}$ in Eigen vector regarding each row. The method to get the CM vector Yj is indicated in Equation (8). The CM vector is computed to be an average for computing $\lambda_{\max}$, as shown in Equation (9). To obtain $\lambda_{\max}$, matrix (4) is normalized by using the expression (5). Next, the eigenvector is calculated through expression (6). Furthermore, expression (7) and 8 are used to obtain Y_j_. Finally, $\lambda_{\max}$ is derived from Y_j_ vector according to expression (9). The ${\hat{h}}_{i}$ values for expression (7) are ${\hat{h}}_{1}=$0.0887, ${\hat{h}}_{2}=$0.1914, ${\hat{h}}_{3}=$0.1914, ${\hat{h}}_{4}=$0.0446, ${\hat{h}}_{5}=$0.4390, ${\hat{h}}_{6}=$0.0446. These values are obtained using expression (5), and 6. We then compute Y_j_ vector as Y_1_ = 6.427, Y_2_ = 6.478, Y_3_ = 6.478, Y_4_ = 6.340, Y_5_ = 6.454, Y_6_ = 6.340 by using expression (7). In expression (7), values were put from expression (4), and (6). Then, $\lambda_{\max}=6.419$ according to expression (9) by inserting the values of Y_j_ from expression (7) and (8).

(3) $${\left[\begin{array}{ccccc} 1 & a_{12} & a_{13} & \to & a_{1n}\\ \frac{1}{a_{12}} & 1 & a_{23} & \to & a_{2n}\\ \frac{1}{a_{13}} & \frac{1}{a_{23}} & 1 & \to & a_{3n}\\ ↓ & ↓ & ↓ & 1 & ↓\\ \frac{1}{a_{1n}} & \frac{1}{a_{2n}} & \frac{1}{a_{3n}} & \to & 1 \end{array}\right]}_{\;}$$

(4) $${\left[\begin{array}{cccccc} 1 & \frac{1}{3} & \frac{1}{3} & 3 & \frac{1}{5} & 3\\ 3 & 1 & 1 & 5 & \frac{1}{3} & 5\\ 3 & 1 & 1 & 5 & \frac{1}{3} & 5\\ \frac{1}{3} & \frac{1}{5} & \frac{1}{5} & 1 & \frac{1}{5} & 1\\ 5 & 3 & 3 & 9 & 1 & 9\\ \frac{1}{3} & \frac{1}{5} & \frac{1}{5} & 1 & \frac{1}{9} & 1 \end{array}\right]}_{\;}$$

(5) $${\left[\begin{array}{ccc} \frac{a_{\left(1,1\right)}}{\sum_{i=1}^{n}a_{\left(i,1\right)}} & \cdots & \frac{a_{\left(1,n\right)}}{\sum_{i=1}^{n}a_{\left(i,n\right)}}\\ \vdots & \ddots & \vdots \\ \frac{a_{\left(n,1\right)}}{\sum_{i=1}^{n}a_{\left(i,1\right)}} & \cdots & \frac{a_{\left(n,n\right)}}{\sum_{i=1}^{n}a_{\left(i,n\right)}} \end{array}\right]}_{\;}$$

(6) $${\hat{H}}_{i}=\frac{1}{n}\sum_{j=1}^{n}a_{\mathrm{ij}}\;\mathrm{where}\;i=1,2,3,\ldots ,n$$

(7) $$\left[\begin{array}{c} Y_{1}\\ Y_{2}\\ Y_{3}\\ ↓\\ Y_{n} \end{array}\right]=\left[\begin{array}{ccccc} a_{11} & a_{12} & a_{13} & \to & a_{1n}\\ a_{21} & a_{22} & a_{23} & \to & a_{2n}\\ a_{31} & a_{32} & a_{33} & \to & a_{3n}\\ ↓ & ↓ & ↓ & ↓ & ↓\\ a_{n1} & a_{n2} & a_{n3} & \to & {\;a}_{\mathrm{nn}} \end{array}\right]\times \left[\begin{array}{c} {\hat{h}}_{1}\\ {\hat{h}}_{2}\\ {\hat{h}}_{3}\\ ↓\\ {\hat{h}}_{n} \end{array}\right]$$

(8) $$Y_{j}=\frac{R_{j}\times {\hat{H}}_{i}}{{\hat{h}}_{i}}\;\mathrm{where}\;j=1,\;2,\;3,\;\ldots ,\;n$$

(9) $$\lambda_{\max}=\frac{1}{n}\sum_{j=1}^{n}Y_{j}$$

### 4.3. Finding the Consistency Index

Consistency Index (U): The U signifies the divergence in consistency [48] of a component’s pairwise comparison matrix. Equation (10) is used to get the U of the pairwise comparison matrix for the B_1_ criterion by inserting the $\lambda_{\max}$ value from Equation (9). Using Equation (9), the $\lambda_{\max}=6.419$. In Equation (10), n shows the order of the comparison matrix in the controller selection. Herein, six alternatives or controllers are compared with each other using a 6 × 6 matrix, therefore n is equivalent to 6. Using Equation (10), a value of 0.0839 was obtained for U.

(10) $$U=\frac{\left(\lambda_{\max}-n\right)}{\left(n-1\right)}$$

Consistency Ratio (K): The K value is used to determine the dependability of the pairwise comparison matrix. Equation (11) is used to compute the value of K. It provides the information about the judgments made in the pairwise comparison matrix. i.e., if these are consistent or not. Hence the condition is K ≤ 0.10 for consistent judgments. For example, if we make the pairwise judgments in the comparison matrix and the K = 2, then it means that there is inconsistency in our judgments. In the pairwise comparison matrices, we compare the controllers regarding their features. For example, which controller is having good support for OpenFlow with respect to others, i.e., we compare controller 1 against all other controllers, then we compare controller 2, 3, 4, 5, and 6 against others. Hence, if we mention in the comparison matrix that controller 1 is better than controller 2 with regard to this feature (OpenFlow), then in the next comparison we give high priority to controller 3 as compared to controller 2, therefore in the subsequent comparison we say that controller 1 has less priority than controller 3. This will raise inconsistency in judgments, and it will not satisfy the condition for the K value, i.e., K will be not less than or equal to 0.1 in this case.

**Figure 3:**
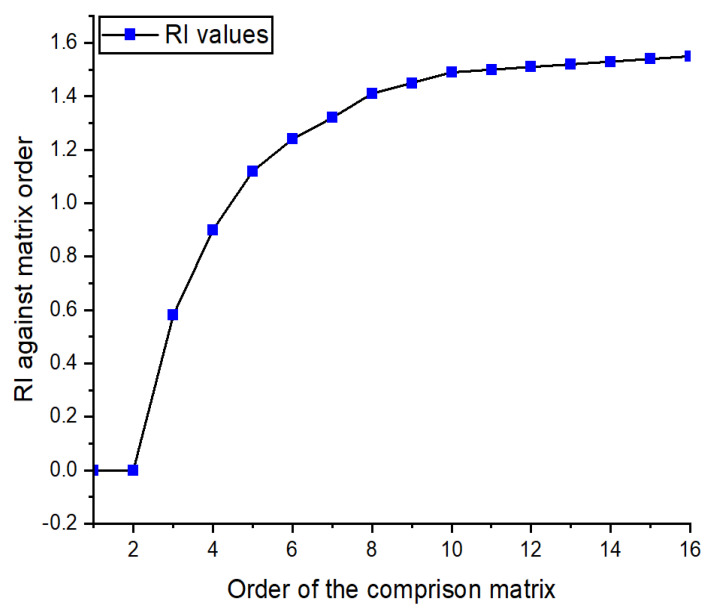
The order of the matrix vs. RI values [46].

The index ratio is denoted by the ratio index (RI) in Equation (11). Table 4 yields the value RI = 1.24 constructed by observing the order of matrix (3). If the matrix’s rank is 3 (the controllers under consideration), a value equivalent to three is chosen for RI. In our assessments, the controller’s number under evaluation in this scenario is six. As a result, the value 6 from Table 4 will be added as mentioned in [48]. In this case n = 6. RI is the consistency index of the random reciprocal matrix generated from the 5-level scale. For the order of matrix greater than 9, the values for RI are approximately leveled with negligible difference, as shown in Figure 3. Furthermore, in the paper [55], the researchers have proposed how to find the RI for with matrix whose order is greater than 9. Finally, the K is calculated by plugging the U value from Equation (10) into Equation (11). From Equation (10), U = 0.0839, RI corresponding to n = 6 in Table 4 is 1.24.

(11) $$K=\frac{U}{\mathrm{RI}}$$

The K = 0.067 according to operations performed in Equation (11). Herein, the K value satisfies the condition i.e., K ≤ 0.10 because the value for K we have calculated is 0.067. The controllers are pairwise compared for remaining features i.e., B_2_->B_10_. The U and K values are computed using the same method for remain matrices. The K value is confirmed for the six controllers. The eigenvectors relating to Bi, is $\hat{H}$i, where $\hat{H}$_1_ signifies the eigenvector equivalent to the B_1_ criterion. Likewise, $\hat{H}$_2_ reveals the eigenvector for B_2_ feature, $\hat{H}$_3_ for B_3_ etc. The next phase in the ANDP model is to find the unweighted and weighted super-matrices to obtain the resultant significant controller listing.

### 4.4. Calculation of the Final Controller Weights

The eigenvectors produced in comparison matrices (which reveal the weight of each criterion with regard to every one option (controller) and vice versa) are merged and expressed in an unweighted super-matrix (USM). Next, the USM is modified to be column stochastic, with the total of column fields in the matrix are made equivalent of one. After this, the matrix is transformed into a weighted super-matrix as a result of this activity (WSM). The WSM and USM are the same thing. The sole distinction between them is that the WSM is column stochastic. In Table 5, D_1_–D_6_ reflect the priority values of the options (controllers) with respect to each characteristic. The computation of the limit super-matrix is the next step in the ANDP model to acquire the final stable ranking of the controllers.

The WSM must be handled by increasing the power of the matrix until it converges to the fixed values for controllers, known as the limit-super-matrix (LSM). The LSM indicates the weights of the controllers ranked regarding features significant for SD-IoT. Likewise, LSM indicates the final weights quantified against each factor in the criterion and alternative clusters. It is derived from WSM in which the values are raised as power of 2*k* in order to acquire an equal value against each row in LSM [22], where *k* denotes a random integer. The LSM aggregates all matrices’ pairwise comparisons. Table 5 shows LSM, with greater values representing the standing alternative. It shows that D3 has the greatest weights, indicating that it is the best controller for SD-IoT. The resulting alternative weights are shown in Table 5. D3 has a high weight value, hence this SDN controller is optimal according to these weights from LSM. As a result, this D3 controller’s experimental performance is evaluated in the next section for SD-IoT.

## 5. Simulation Setup, Results and Discussion

In this section, we explain the experimental setup to evaluate the performance of the controllers for SD-IoT. Moreover, the traffic generation mechanism is illustrated in detail with steps on the sending and receiving hosts. More and more, the performance evaluation metrics are discussed for the proposed scheme and the previous approaches [24,25].

### 5.1. Experiment Software and Network Infrastructure for SD-IoT

The Mininet emulator with python-based API was used to make the SDN physical architecture on the three controllers, as calculated by using the suggested ANDP, EB-TOPSIS [25] and AHP [24] approaches. This network emulator is often used for prototyping SDN-based experiments. In our testbed we have installed the Ubuntu 16.04 LTS and Mininet version 2.3.0d1. In addition, an OVS switch version of 2.5.4 was installed. Moreover, the Xming utility was executed on the hosts to create and display traffic between the source and target hosts in the network. We generated topologies of sensor nodes up to 500 in Mininet by increasing the number of nodes in a linear physical architecture and collected performance data for each controller under study.

### 5.2. Experimental Scenarios and Traffic Generation Parameters

In the first experiment, we calculate the delay in two scenarios i.e., (1) when the sensor nodes direct traffic towards the controller (Packet-In messages) managing the nodes is uniform, and (2) when the sensor nodes generate traffic and send it to the controller i.e., in the case of new packets arriving at the controller. The distributed Internet traffic generator (D-ITG) [56] has been applied for traffic origination between source and target hosts in each sensor nodes network i.e., SD-IoT. The stepwise process for generating of traffic between hosts in the sensors network is explained in Algorithm 1. The Algorithm 1 shows that we open the two terminals on the sending and receiving hosts in the network of sensor nodes (ranges from 1–500 nodes). The packets are generated using the parameters mentioned in the step 5 of algorithm 1. If the flow entry for a packet is not present in the flow table, then it is sent as Packet-In message to the controller. Hence, the packets are sent continuously towards the SDN controller.

A listening socket is forked on the target host for the transmission control protocol (TCP) communication from source nodes through ITGRecv (H2). We selected class C IP addresses for the entire network, and ITGSend is used on the source host (H1) to transmit TCP traffic with a payload of 5000 bytes for 1000 s (sec) at a rate of 10,000 packets/sec to a destination node having the IP address 192.168.1.10. The experiment was repeated 10 times, and the average findings for delay are displayed in the figures below.


**Algorithm 1: Traffic Generation Algorithm**
Step 1: Open the graphical terminal of the H2Step 2: On the host H2, change the directory to D-ITG/binStep 3: Execute the command: /ITGRecv on the terminal of H2Step 4: In addition, open the GUI terminal of H1 hostStep 5: Type the command: /ITGSend -T TCP -A 192.168.1.19 -c 5000 -C 10,000 -t 1000 -l sender.log -x receiver.logStep 6: Traffic log analysis commands on H1, and H2Step 7: H1: /ITGDec sender.logStep 8: H1: /ITGDec receiver.log

### 5.3. Delay Comparison for SD-IoT

**Figure 4:**
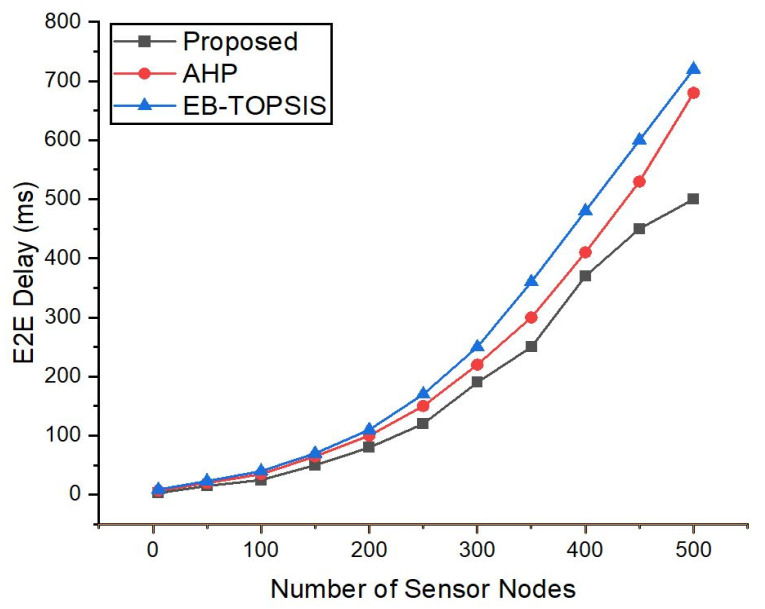
Delay recorded with increasing the number of sensor nodes.

Figure 4 shows the delay between source and destination hosts that we attached to the sensor nodes in Mininet. Figure 4 reveals that with an increasing number of IoT sensor nodes the delay between source and destination hosts is increasing. The results imply that the delay evaluated in the continued traffic between hosts (source-to-destination) for our suggested controller is reduced more than for the AHP controller as well as in the EB-TOPSIS scheme. This is due to a fast response to the flow requests and the scalability features of the proposed controller. Hence, we can see that the delay is less when compared to the previous methods because during the selection of a controller for SD-IoT these features contributed less for the AHP controller. Therefore, the features of the ANDP controller contributed to the reduction in delay.

### 5.4. Delay Evaluation for SD-IoT Amid Traffic Generation

**Figure 5:**
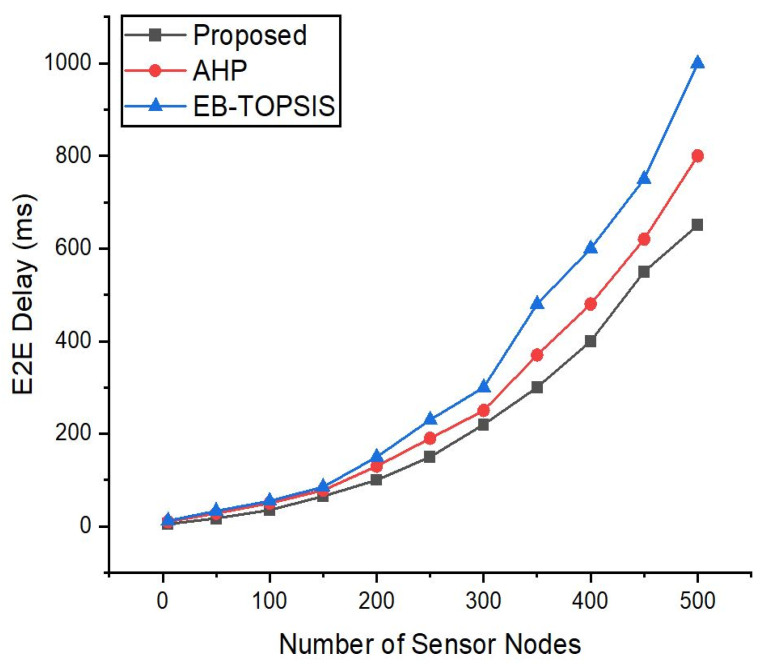
Delay recorded while traffic generation and increasing the sensor nodes.

Figure 5 shows the delay recorded under the traffic generation scenario and scalability. Herein, Figure 5 shows that with an increasing number of IoT sensor nodes and traffic, the delay between the source and target hosts is increasing. The results reveal that the delay generated due to high traffic generation with the proposed controller is smaller than the controller computed through the AHP mechanism. This is due to the delay reduction in flow requests management and load balancing features for the proposed controller as well as the flow requests’ fast response capability. Therefore, the graph shows promising results in terms of delay even with the heavy traffic load that we generated through D-ITG for the proposed controller in contrast to the benchmark model and EB-TOPSIS strategy.

### 5.5. Throughput Analysis

**Figure 6:**
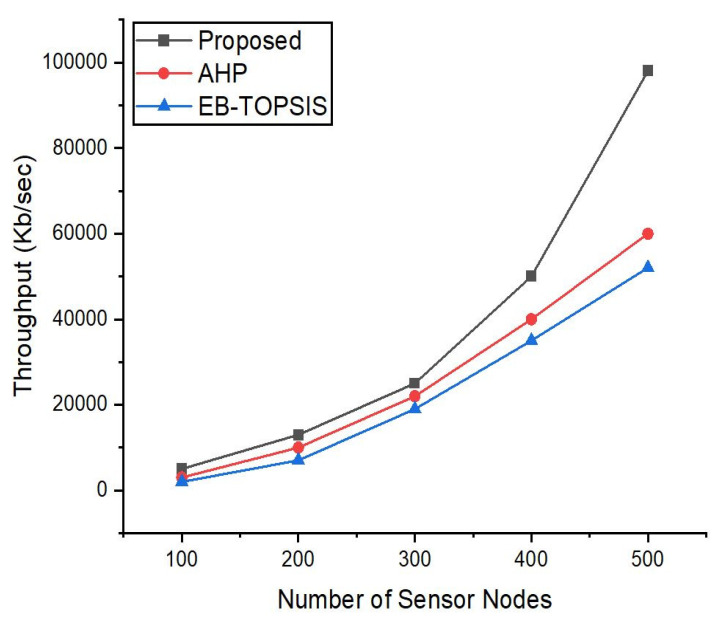
Throughput evaluation of the proposed scheme and previous approach.

Figure 6 compares the throughput of three controllers calculated using our suggested ANDP approach controller and two other approaches. Herein, Cbench tool [5] was used to compute the throughput by delivering Packet-In messages controllers (these messages are forwarded towards the controller in case of a table miss or when there is no flow rule in the attached switches to the controller) and computing the number of Packet-Out (responses/second). The MACs simulated per switch were fixed at 5000 in this case. However, the number of nodes were increased to 500, and each test was repeated ten times. Using Cbench, we defined these parameters for MACs upon the nodes, and they range from 100 to 500. Furthermore, we tested the throughput with the three controllers selected with the proposed method, AHP and EB-TOPSIS, and plot the results of the throughput for each scheme. The average findings reveal that the suggested controller’s throughput does not decline and has a faster start than the controller obtained via benchmark studies. Figure 6 indicates that with an increase in the number of nodes this rate of throughput is higher for the proposed controller as compared to the other two controllers.

### 5.6. CPU Utilization Analysis

**Figure 7:**
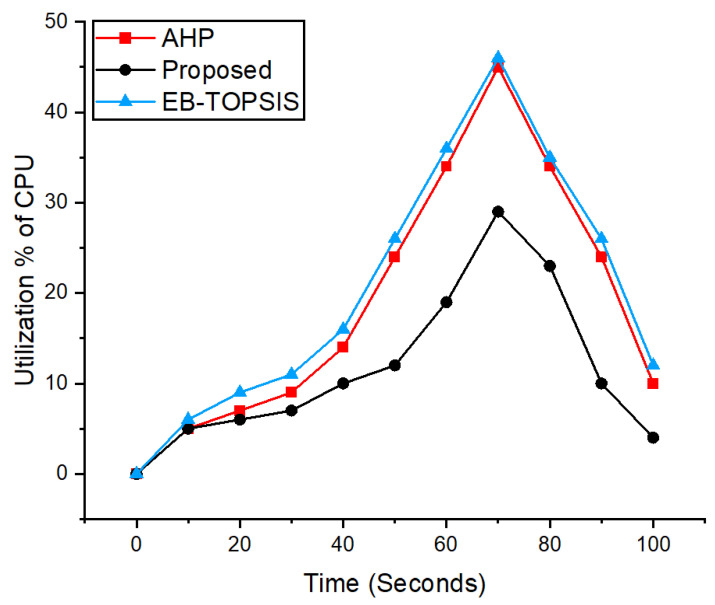
CPU utilization analysis of the proposed method and previous scheme.

Figure 7 depicts the CPU use during traffic creation for the experiment as measured by Sysbench software [57], while evaluating the three controllers, i.e., the AHP-selected controller, EB-TOPSIS, and the suggested technique, during traffic generation. The parameters of the traffic generation are the same as we provided in algorithm 1 regarding the packet size and the number. We have plotted the results for 100 s emulation time, which is given on the *x*-axis. The *y*-axis denotes the percentage of CPU utilization. The experiment is performed using the Mininet tool [6] for the three controllers. The graph indicates that the proposed scheme has a better CPU utilization percentage as compared to the AHP and EB-TOPSIS controllers. Therefore, the controller utilizes the resources efficiently during the traffic generation experiment. This shows that the proposed scheme can perform efficiently even with an increase in the traffic. The resultant significant results were achieved due to the selection of the IoT features in the desired ANDP controller.

### 5.7. Reliability Evaluation

**Figure 8:**
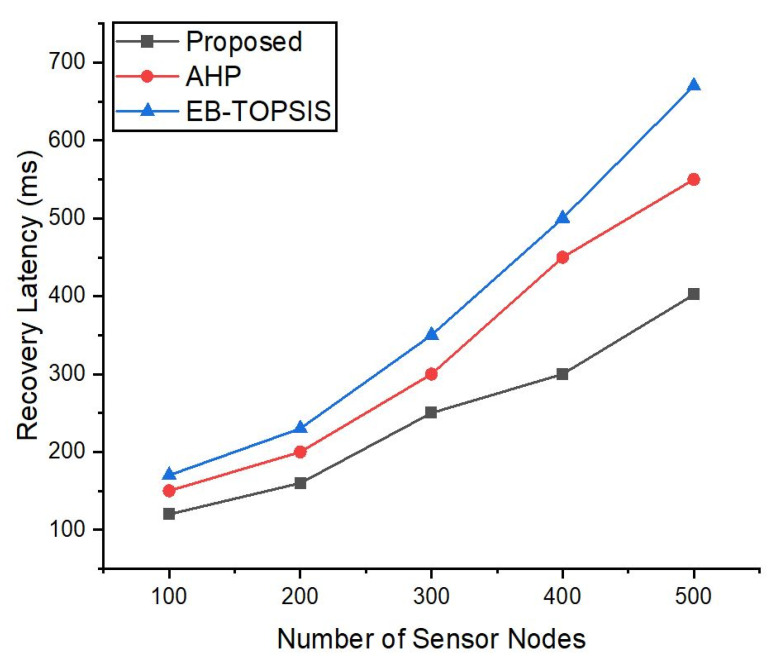
Link failure recovery latency comparison of the proposed framework with AHP and EB-TOPSIS approaches.

Figure 8 shows an evaluation of recovery times (shown in milliseconds (ms)) intended for a link failure in the network [58] for the ANDP and benchmark methods with the Onos, Floodlight and Ryu controllers. To simulate the experiment, we made a failed link in Mininet with a link down command and recorded the total latency of recovery for the Onos, Ryu, and Floodlight controllers in the SDN they took to recover the network to an operational state. The total recovery latency denoted with *LR_total_* is obtained using Equation (12). Herein, *DF* denotes failure detection latency (ms), *PC* indicates path calculation latency (ms) i.e., for computing the alternate path, and *FI* shows a flow installation latency (ms). Figure 8 shows that the recovery latency in the ANDP controller is smaller compared to the AHP controller. Furthermore, the recovery latency for Floodlight and Ryu is significantly higher than for Onos, since it supports the significant features which play a role in faster link recovery.

(12) $$LR_{total}=\left(L_{FD}+L_{PC}+L_{FI})\right.$$

## 6. Conclusions

The goal of this research was to choose the optimal SDN controller, based on its features and performance, for SD-IoT. It is considered an MCDM issue since the controller selection procedure was based on several aspects such as platform compatibility, NB-API, SB-API, scalability, and flow request processing capabilities, etc. As a result, the proposed ANDP method was employed to tackle this issue. The goals were specified initially, followed by criteria based on which controller must be picked and the alternatives to be prioritized for selection. Following that, a pairwise comparison was made using a matrix to compare each element (controller) in the criteria (features) cluster with each option in the alternative cluster i.e., a set of six controllers. The ultimate outcome matrix, referred to as an LSM, prioritizes the controller. As a result, a controller possessing high weight was chosen for additional quantitative study of SD-IoT experiments and a comparison was made with benchmark models. The findings of the LSM revealed that the D3 controller has the best features for SD-IoT. To validate the performance of the three controllers obtained through the proposed approach, a quantitative experimental analysis of the three controllers was conducted, which included assessing the QoS parameters, such as delay with and without traffic generation, CPU usage, throughput and the failure recovery latency. D3 outperforms the D5 and D1 controllers in experimental assessments according to the experimental data confirmed by Mininet, and therefore it is indicated that it is the best controller for SD-IoT.

## Figures and Tables

**Table 1:** List of features for SD-IoT performance evaluation.

Serial#	Name	Notation	Description
1	OpenFlow-support	B_1_	OpenFlow 1.0–1.5
2	GUI	B_2_	Web based or Python based
3	NB-API support	B_3_	REST-API.
4	Clustering support	B_4_	To ensure reliability and performance
5	Openstack networking	B_5_	Enabling different network technologies via quantum API
6	Synchronization	B_6_	State synchronization of the clusters
7	Flow requests handling	B_7_	The capability to handle the flow requests
8	Scalability	B_8_	Adoptability in the extended networks
9	Platform support	B_9_	Windows, Mac, Linux
10	Efficient energy management	B_10_	The ability of efficient energy utilization

**Table 2:** List of controllers for comparison and notations.

Serial#	1	2	3	4	5	6
Name of controller	Floodlight	Odl	Onos	Pox	Ryu	Trema
Notation	D_1_	D_2_	D_3_	D_4_	D_5_	D_6_

**Table 3:** Features classification levels in the controllers for SD-IoT.

Controllers	Features									
	B_1_	B_2_	B_3_	B_4_	B_5_	B_6_	B_7_	B_8_	B_9_	B_10_
D_1_	G_2_	G_4_	Yes	Yes	No	G_2_	G_2_	G_1_	G_1_	G_2_
D_2_	G_3_	G_3_	Yes	Yes	Yes	G_2_	G_2_	G_4_	G_3_	G_3_
D_3_	G_3_	G_3_	Yes	Yes	Yes	G_3_	G_4_	G_4_	G_3_	G_4_
D_4_	G_1_	G_2_	No	No	No	G_1_	G_3_	G_1_	G_3_	G_1_
D_5_	G_4_	G_1_	No	Yes	No	G_3_	G_3_	G_2_	G_1_	G_3_
D_6_	G_1_	G_1_	No	Yes	No	G_1_	G_3_	G_1_	G_1_	G_2_

**Table 4:** Ratio index used for various number of features and controllers [48].

Comparison Matrix Order	RI Value
1	0.00
2	0.00
3	0.58
4	0.90
5	1.12
6	1.24
7	1.32
8	1.41
9	1.45
10	1.49

**Table 5:** Ranking of the controller for SD-IOT using ANDP.

Controller	Weightage
D1	0.049
D2	0.078
D3	0.110
D4	0.039
D5	0.099
D6	0.029

## Data Availability

Not applicable.
